# Phylogeographic Structure and Population Dynamics of Baoxing Osmanthus (*Osmanthus serrulatus*), an Endemic Species from the Southwest Sichuan Basin, China

**DOI:** 10.3390/plants13121653

**Published:** 2024-06-14

**Authors:** Zhibei Wang, Xi Wu, Xin Liu, Min Zhang, Chunping Xie, Lin Chen

**Affiliations:** 1 Co-Innovation Center for the Sustainable Forestry in Southern China, College of Life Sciences, Nanjing Forestry University, Nanjing 210037, China; august08@njfu.edu.cn (Z.W.); scarlet@njfu.edu.cn (X.W.); lxxmv67@163.com (X.L.); zhangmin@njfu.edu.cn (M.Z.); 2 International Cultivar Registration Center for Osmanthus, Nanjing Forestry University, Nanjing 210037, China; 3 Tropical Biodiversity and Bioresource Utilization Laboratory, Qiongtai Normal University, Haikou 571127, China; xcp@mail.qtnu.edu.cn

**Keywords:** *Osmanthus serrulatus*, cpDNA, genetic structure, phylogeography, population dynamics

## Abstract

The mountainous regions of southwest China are recognized as pivotal centers for the origin and evolution of *Osmanthus* species. Baoxing Osmanthus (*Osmanthus serrulatus* Rehder), a rare and endemic species known for its spring blooms, is sparsely distributed within the high altitude evergreen broad-leaved forests surrounding the southwestern Sichuan Basin. However, persistent anthropogenic disturbances and habitat fragmentation have precipitated a significant decline in its natural population size, leading to the erosion of genetic resources. To assess the genetic status of *O. serrulatus* and formulate effective conservation strategies, we conducted sampling across ten wild populations, totaling 148 individuals in their natural habitats. We employed two cpDNA fragments (*matK* and *trnS-trnG*) to elucidate the phylogeographic structure and historical population dynamics. The results revealed low species-level genetic diversity, alongside pronounced regional differentiation among populations (*F_ST_* = 0.812, *p* < 0.05) and a notable phylogeographic structure (*N_ST_* = 0.698 > *G_ST_* = 0.396, *p* < 0.05). Notably, genetic variation was predominantly observed among populations (81.23%), with no evidence of recent demographic expansion across the *O. serrulatus* distribution range. Furthermore, divergence dating indicated a timeline of approximately 4.85 Mya, corresponding to the late Miocene to early Pleistocene. This temporal correlation coincided with localized uplift events in the southwestern mountains and heightened Asian monsoons, suggesting pivotal roles for these factors in shaping the current phylogeographic pattern of *O. serrulatus*. These findings support the effective conservation of *O. serrulatus* germplasm and offer insights into the impact of Quaternary climate oscillations on companion species within evergreen broad-leaved forests. They also enhance our understanding of the origin and evolution of these forests in the southwestern mountains, aiding biodiversity conservation efforts in the region.

## 1. Introduction

The southwestern region of China, especially the eastern Himalayan and Hengduan Mountain areas of the Qinghai–Tibetan Plateau, is renowned for its wide variety of plant species and a high proportion of endemic plants [1,2]. This region is the most biodiverse in China and is a critical center for global biodiversity [3]. The uplift of the Qinghai–Tibetan Plateau and drastic changes in East Asian climate increased the thermal contrast between the ocean and the land, fostering the development of the Asian monsoon system [4]. These conditions favoured the expansion of moist forests, creating geographically and ecologically isolated habitats that facilitated the diversification in and around the Tibetan Plateau [5].

Climate change has a profound impact on the formation and distribution of organisms, and the current geographical distribution of plants roughly reflects their adaptation to climate fluctuations in the past glacial ages [6,7]. Glacial refugia, areas not covered by ice sheets during glacial periods, offered sanctuaries for the survival and reproduction of many species. Unlike the relatively singular and concentrated refugia in Europe and America, China’s minimal glaciation impact led to a lack of common geographical barriers among species, resulting in heterogeneous phylogenetic structures and population histories with limited post-glacial expansion and no clear migration routes [8,9,10]. This led to varied and dispersed glacial refugia, such as the mountains in western Sichuan, part of the eastern extension of the Tibetan Plateau, and the Hengduan mountain range. The complex terrain, significant altitude differences, and diverse climate of this region provided rich growing conditions for plants, accelerating inter-population differentiation [8,11]. Populations located in refugia often exhibit high levels of genetic diversity, haplotype polymorphism, and endemism. This could be attributed to the haplotype preservation or new haplotype formation during post-glacial species migration and expansion [12,13]. Therefore, the richness and phylogenetic relationships of these haplotypes may be closely related to their geographical environments. Analyzing haplotype diversity and its geographical distribution can infer the origins of species and the phylogenetic relationships between populations, thereby determining their evolutionary pathways.

The complex and diverse phylogeographic patterns of different species in the Qinghai–Tibet Plateau and its surrounding areas are influenced by multiple factors. The uplift of the Qinghai–Tibet Plateau, intense climate fluctuations during the Quaternary glaciations, and biological differences among species in pollen and seed dispersal collectively result in significant differences in the speciation and differentiation times of different species in this region [14,15,16]. Additionally, the complex terrain divides species into small populations in different areas, where limited gene flow leads to pronounced phylogeographic structures [11]. This is often manifested as a relatively uniform haplotype on the Qinghai–Tibet Plateau and high haplotype diversity in the southeastern and adjacent areas [17].

Sweet Osmanthus, collectively known as the plants of genus *Osmanthus*, is one of the top ten traditional flowers in China. It has a long cultivation history and cultural significance, serving important economic roles in ornamental, aromatic, culinary, and medicinal applications [18]. The genus *Osmanthus* comprises 27 species, with all but one (*O. decorus*, which is found in the Caucasus region) primarily distributed in East Asia [19]. Among them, there are 21 species widely distributed in mountainous areas from eastern to southwestern China, making them an important companion species in the subtropical evergreen broad-leaved forests of China [18]. The southwestern mountains may be one of the crucial centers for its origin and evolution [18,19]. *Osmanthus* is a typical polyphyletic group with species that lack distinct morphological characteristics for differentiation [19,20]. The sweet osmanthus can be roughly divided into two types, based on their flowering periods in spring or autumn. The autumn-flowering types are mostly distributed in the hilly and mountainous regions of southeast China, while the spring-flowering types are primarily found in the high-altitude mountainous regions of southwest China. Baoxing Osmanthus (*Osmanthus serrulatus* Rehder), a rare and endemic wild sweet osmanthus blooming in spring, is sparsely distributed in the narrow habitats of high-altitude mountains around the southwestern Sichuan Basin, holding significant economic and scientific value [18,21]. The geographical distribution of *O. serrulatus* is contested. The Flora of China (FOC) documents this species in Guangxi and Fujian, while current evidence indicates that the species in Guangxi is *O. reticulatus* and in Fujian, it is *O. henryi* [22]. Although morphologically similar, *O. reticulatus* and *O. henryi* bloom in autumn, whereas *O. serrulatus* blooms in spring, facilitating their distinction [22,23]. No specimens have been found in Guangxi and Fujian during our field surveys over the past decade, confirming that *O. serrulatus* is restricted to the mountainous regions of southwestern Sichuan.

Habitat fragmentation and overexploitation have severely threatened the wild resources of *O. serrulatus* in recent years, leading to increasing genetic erosion [24]. Our previous work clarified its breeding system, seed germination, habitat characteristics, genetic diversity, and population structure, highlighting the compound threats posed by both external and internal factors [25]. The combination of narrow ecological adaptability, difficulty in natural regeneration, habitat fragmentation and persistent and serious human interference pose a critical extinction risk to its wild populations [19,21,24,25,26,27,28]. Here, guided by the hypotheses of (1) geographic and climatic factors have shaped the phylogeographic pattern of *O. serrulatus*, and (2) the southwestern mountainous regions served as crucial refuges for *O. serrulatus* during the Quaternary glacial glaciations, with geographic barriers limiting the distribution and expansion of its populations, we utilize two maternally inherited chloroplast DNA (*matK*, *trnS-G*) sequences to refer to the phylogeographic structure and population dynamics of *O. serrulatus*, aiming to provide a scientific basis for its conservation and utilization. Furthermore, we seek to offer new insights into the response and evolutionary processes of companion species in subtropical evergreen broad-leaved forests to climate changes in the southwestern mountains, thereby elucidating the patterns and mechanisms behind the biodiversity of China’s subtropical forests.

## 2. Results

### 2.1. Sequence Variation and Genetic Diversity

The sequencing data of amplified products for two chloroplast fragments (*matK* and *trnS-G*) from 148 *O. serrulatus* individuals revealed two and three polymorphic sites, respectively, with a total aligned length of 2059 bp. This included 958 bp of *matK* and 1101 bp of *trnS-G*. In 148 individuals across ten populations, six haplotypes (H1–H6) were identified (Table 1), with H1 and H6 being unique. The most common haplotype, H3, appeared in 90 individuals. Out of the ten populations, two had only one haplotype (DLS2, XLXS), while the others had two to three haplotypes, with the DLS1 featuring three different haplotypes (Table 2).

At the species level, the haplotype diversity (*H*_d_) was 0.590 and nucleotide diversity (π) was 0.47, which suggests a relatively high genetic diversity of *O. serrulatus*. At the population level, *H*_d_ ranged from 0.000 to 0.712, and π ranged from 0.00 to 0.43 (Table 2), suggesting a significant variation in genetic diversity across different populations. The DLS1 exhibited the highest genetic diversity (*H*_d_ = 0.712, π = 0.43), with seven other populations also showing relatively high levels of haplotype and nucleotide diversity, mostly exceeding the average values (*H*_d_ = 0.344, π = 0.17). In contrast, the DLS2 and XLXS had no genetic diversity, with XLXS possessing a unique haplotype.

### 2.2. Genetic Differentiation and Population Structure

Analysis of molecular variance (AMOVA) indicated a high level of population genetic differentiation (*F*_ST_ = 0.812, *p* < 0.05) in *O. serrulatus*, while a low gene flow (*N*_m_ = 0.11) also validated a high genetic differentiation between populations. Genetic variation occurred mainly between populations (81.23%) and only 18.77% within populations (Table 3). Furthermore, the number of substitution types (*N*_ST_ = 0.698) significantly exceeded population differentiation (*G*_ST_ = 0.396, *p* < 0.05), highlighting a clear phylogeographic structure for this endemic species, with low chloroplast haplotype similarity and distinct differentiation among populations.

### 2.3. Distribution Pattern and Phylogenetic Relationship

**Figure 1:**
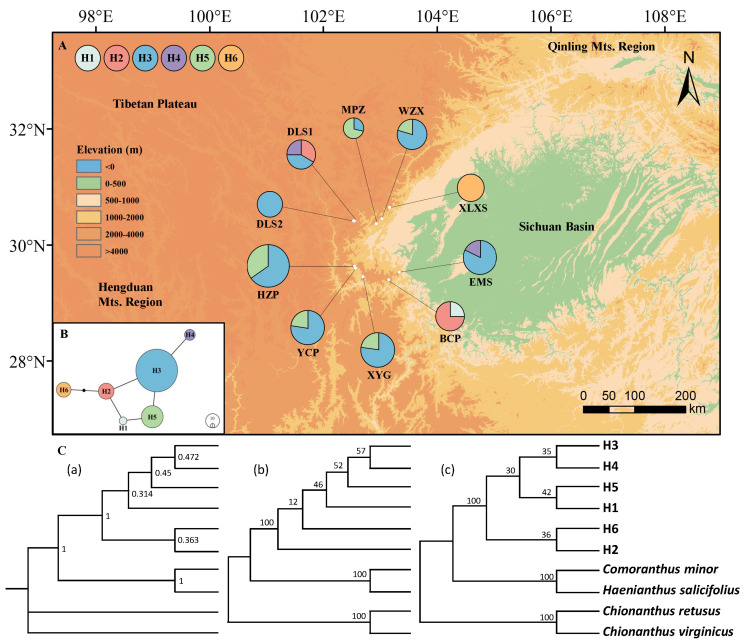
Haplotype structure of cpDNA sequences. (**A**) Geographical distribution map of six chloroplast haplotypes (H1–H6) detected in ten *O. serrulatus* populations. Size of each circle represents the population size; the color of the proportion in a circle indicates the type of haplotype, and the proportion corresponds to the number of individual(s) who have(s) the haplotype. (**B**) The haplotype network diagram of *O. serrulatus*. Each numbered circle (H1–H6) represents a unique haplotype, and the circle size of each haplotype is proportional to its frequency. (**C**) Phylogenetic trees of cpDNA haplotype of *O. serrulatus* based on Bayesian inference (**a**), maximum likelihood (**b**), and neighbor-joining (**c**).

A total of six cpDNA haplotypes of *O. serrulatus* were detected (Figure 1A, Table 1), of which H1 and H6 were endemic to BCP and XLXS, respectively. The other haplotypes were shared across the populations, with the most common haplotype H3 accounting for 80% of populations. The following haplotype H5 was distributed in five populations, including two (WZX, MPZ) in Lushan County and three (HZP, XYG, YCP) in Hanyuan County. The haplotype H2 was only detected in DLS1 and BCP, while H4 was shared by DLS1 and EMS. According to the cpDNA haplotype network diagram (Figure 1B), H3 was located in the central region, while H3 represented a significant number of individuals. Consequently, H3 was inferred to be an ancient haplotype, whereas the remaining haplotypes were considered to be derived.

Using *Chionanthus retusus*, *C. virginicus*, *Haenianthus salicifolius*, and *Comoranthus minor* as outgroups, the phylogenetic trees based on six haplotypes were constructed by Bayesian inference (BI), maximum likelihood (ML), and neighbor-joining (NJ) methods, which exhibited a relatively consistent topological structure (Figure 1C). The phylogenetic trees prominently clustered the haplotypes H1–H6 and haplotypes H1 and H5 formed closely related branches, consistent with the results from the POPART haplotype network diagram.

### 2.4. Divergence Time Estimation and Population Dynamics

**Figure 2:**
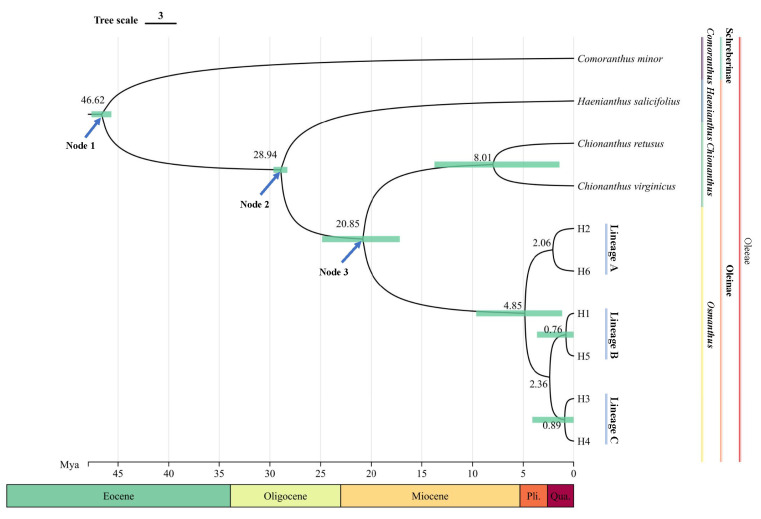
BEAST-derived chronogram for *O. serrulatus* based on concatenated cpDNA (*matK* and *trnS-trnG*). Ages of Node 1, Node 2, and Node 3 have been calibrated, and six haplotypes clustered into three lineages. The divergence times (millions of years ago, Mya) of nodes are shown above branches, and green bars indicate 95% HPD credibility intervals for each divergence time.

The divergence time of the most recent common ancestor (TMRCA) of *O. serrulatus* was estimated to be around 20.85 Mya (95% HPD: 16.14–24.96 Mya), near the early Miocene (Figure 2). The diversification of the six cpDNA haplotypes for *O. serrulatus* was around 4.85 Mya (95% HPD: 1.31–9.64 Mya), corresponding to a geological historical period ranging from the late Miocene to the early Pleistocene. According to the phylogenetic tree, three lineages of *O. serrulatus* haplotypes can be distinguished: Lineage A diverged around 2.06 Mya, while Lineages B and C separated around 2.36 Mya. The divergence relationships were generally consistent with the phylogenetic trees.

**Figure 3:**
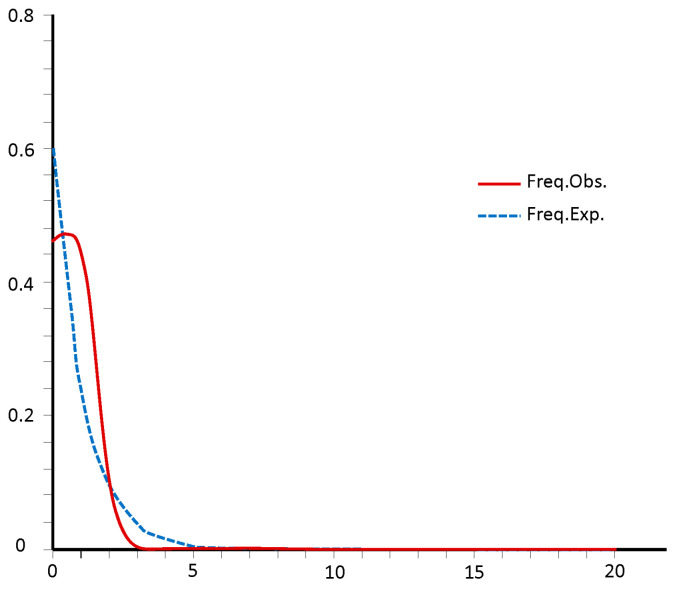
Mismatch distribution for all populations of *O. serrulatus*.

To investigate the historical population dynamics of *O. serrulatus*, a neutrality test and mismatch distribution analysis were performed using chloroplast-associated sequences. In the neutrality tests, all the values of Tajima’s *D* and Fu’s *Fs* were insignificant positive, with an average of Tajima’s *D* = 0.466 (*p* > 0.05) and Fu’s *Fs* = 0.588 (*p* > 0.05) (Table 4). The observed mismatch distributions of the pairwise nucleotide differences exhibited a unimodal distribution for all populations of *O. serrulatus* based on cpDNA, with the values of the sum of squared deviations between observed and expected (*SSD*) and raggedness index (*H*_Rag_) were not significant (*p* > 0.05) (Figure 3, Table 4). All the evidence indicated that no recent demographic expansion occurred in all *O. serrulatus* populations across the distribution regions.

## 3. Discussion

### 3.1. Genetic Diversity and Haplotype Variation

Genetic diversity is essential for species survival, reflecting their capacity to adapt to environmental changes [29]. Adequate genetic diversity is crucial for species to thrive in their natural habitats [30]. Metrics like haplotype diversity (*H*_d_) and nucleotide diversity (π) serve as key indices of genetic diversity, with higher values indicating stronger adaptability and breeding potential [31]. Higher *H*_d_ and π values generally signify better adaptive and survival abilities within populations, offering greater opportunities for breeding and genetic improvement. In this study, *O. serrulatus* exhibited relatively low genetic diversity across its current geographical distribution, with *H*_d_ = 0.590 and π = 0.47, suggesting lower genetic diversity compared to the average chloroplast genetic diversity of 170 reported plant species (*H*_T_ = 0.67) [32].

There was a private haplotype, H1 and H6, detected from the population of BCP and XLXS, respectively. These two populations are located in deep mountains with complex terrain. Isolated by these natural barriers, limited gene exchange and dissemination have led to the emergence of distinct genetic haplotypes. Conversely, populations of HZP, XYG, YCP, WZX, and MPZ, situated on the mountain periphery, benefit from gentler terrain, facilitating gene exchange and dissemination, resulting in uniform haplotypes. Notably, population DLS1, DSL2, and EMS, perched at higher elevations, all exhibit haplotype H4, suggesting its probable association with altitude. Noteworthy findings indicate that, besides the unique haplotypes observed in BCP and XLXS, the majority of haplotypes are shared among populations, with H3 prevailing in 80% of cases, suggesting its potential as an ancient haplotype. These patterns suggest recent fragmentation and bidirectional gene flow within *O. serrulatus* populations, aligning with prior research on community structure [20,21,28].

Habitat fragmentation and degradation induce a decline in population size, further triggering random genetic drift, thereby leading to bottlenecks and the loss of alleles within the species [33]. *O. serrulatus* is sporadically distributed throughout the high-altitude mountains encircling the southwest Sichuan Basin, characterized by complex geographical and climatic conditions and diverse habitats [24]. This region is integral to the “southwest China Sky Island Complex”, where diverse subareas exhibit considerable variability in both topography and climate [11]. Such variability frequently confines *O. serrulatus* populations to isolated and diminutive habitats. Our previous studies have identified several initial characteristics of plant species with extremely small populations (PSESPs) in *O. serrulatus* [24,27]. These characteristics indicate that habitat loss and fragmentation are the primary factors responsible for the decline in population size and genetic diversity [25]. The escalating human-induced destruction and deforestation have exacerbated the fragmentation and degradation of *O. serrulatus* habitats [24]. This habitat fragmentation is compounded by geographic barriers such as altitude and distance, which further accelerate genetic diversity loss and restrict genetic exchange among populations. Consequently, there is high genetic diversity within populations but low genetic differentiation among them [25]. This pattern suggests that genetic erosion and genetic drift are likely to further reduce genetic diversity in subsequent generations. Therefore, habitat fragmentation is likely a major factor contributing to the low genetic diversity observed in *O. serrulatus*.

Chloroplast DNA (cpDNA), characterized by uniparental inheritance, low mutation rates, and haploid, retains historical genetic traces of plant evolution, aiding in understanding the phylogeographic variation mechanisms [34]. The genetic diversity detected in this study (*H*_d_ = 0.590) was lower than our earlier study using SSR markers (*H*_e_ = 0.694) [25], primarily due to the slower evolutionary rate of the maternally inherited chloroplast genes compared to the biparentally inherited nuclear genes [32]. Additionally, this study suggests that *O. serrulatus* is a relatively young species that underwent early differentiation in the early Pliocene (4.85 Mya). Its short evolutionary history and the slower evolutionary rate of chloroplasts may have limited the accumulation of extensive variation [35]. Significant differences exist between the chloroplast genome and the nuclear and mitochondrial genomes regarding structure, inheritance mode, mutation rate, gene flow, and effective population size [34]. These differences determine potential discrepancies in results when analyzing phylogeographic variation using cpDNA compared to nDNA or mtDNA markers. Hence, future comprehensive analyses integrating the genes or DNA fragments with diverse genetic backgrounds and different evolutionary rates, such as cpDNA, nDNA, and mtDNA, will facilitate the dissection of species’ phylogeographic variation at the genomic level. This approach will deepen our understanding of the ecological and evolutionary processes shaping species’ phylogeographical structures and enable the construction of more comprehensive phylogenetic relationships [34,36].

### 3.2. Population Differentiation and Genetic Structure

Geographical isolation and environmental differences are significant drivers of population differentiation within biological communities [37]. High mountains and deep valleys serve as crucial geographic barriers, hindering the long-distance dispersal of pollen and seeds and increasing the likelihood of inbreeding within or between adjacent populations, thereby promoting allopatric differentiation among populations in different geographical regions [38,39]. The wild population of *O. serrulatus* is mainly distributed in the mountainous regions of the southwestern Sichuan Basin, where the alternating high mountains and deep valleys, coupled with the limited seed dispersal ability due to the plant’s seed structure and dispersal mode, restrict gene flow between populations [40]. Limited gene flow exacerbates the effects of genetic drift or directional selection on differentiation, leading to significant genetic divergence and geographical structuring among populations [11,41]. In this study, *O. serrulatus* exhibited high levels of population genetic differentiation (*F_ST_* = 0.812) and significant phylogeographic structure (*N_ST_* > *G_ST_*, *p* < 0.05), indicating the long-term effects of geographical barriers on these wild populations. Additionally, AMOVA analysis revealed that 81.2% of genetic variation occurred among populations, with gene flow estimated at only 0.11, further supporting these findings. The observed genetic patterns underscore the importance of preserving diverse habitats to maintain the genetic diversity and evolutionary potential of this species.

### 3.3. Population Divergence and History Dynamics

Plant species in the southwestern mountains exhibit high diversity and endemism, with many populations showing multiple refugial isolation and long-term demographic stability, making this region a primary refuge for late Pleistocene flora and fauna [42,43]. Molecular clock analyses suggested that intra-specific haplotype differentiation of *O. serrulatus* occurred primarily between the early Pliocene and early Pleistocene, indicating a stable population history contributing to its current geographic distribution [44]. During the late Miocene to Pliocene, intensified Asian monsoons and localized mountain uplifts in the southwestern mountains promoted in situ diversification of many alpine taxa [5,45,46]. Quaternary climatic oscillations likely facilitated species differentiation, with plant populations frequently migrating up and down, and finally isolated in the “sky islands” [43,47]. Therefore, *O. serrulatus* may have survived in situ, retreated during the glacial periods, and undergone limited expansion during the interglacial periods. Haplotype H3, the most common haplotype in the population, emerged during this time. Quaternary climate oscillations further promoted genetic divergence among the populations of *O. serrulatus*. In addition, the divergence time of *O. serrulatus* were consistent with some other species endemic to this region, such as *Sophora davidii* [42] and *Tetrastigma hemsleyanum* [48], which diverged mainly in the Pliocene and persisted into the Pleistocene, suggesting a shared evolutionary history. In this study, the positive values of Tajima’s *D* and Fu’s *Fs* for *O. serrulatus* indicated a deviation from neutrality, while the observed curve did not fit the expected curves in mismatch distribution analysis. Although the *SSD* and *H*_Rag_ values were not significant (*p* > 0.05), when combined with geographical locations, these findings still reflected the past demographic stability of the *O. serrulatus* populations. This stability suggested that despite the lack of significant signs of recent expansions or bottlenecks, the genetic structure of *O. serrulatus* has been influenced by its long-term presence in the region and its ability to persist through climatic fluctuations and geographical changes. All these findings emphasize the importance of considering both geological and climatic factors in understanding the evolutionary dynamics of plant species in the southwestern mountainous region of China.

## 4. Materials and Methods

### 4.1. Population Sampling

Fresh leaf samples were collected from 148 individuals from ten natural populations of *O. serrulatus* in southwestern China, covering its main distribution range (Table 2). There were 7 to 26 individuals per population, depending on the population size, randomly collected within each population, with individuals at least 30 m apart. The collected fresh leaf samples were dried with silica gel and stored at −20 °C. Voucher specimens are stored in the Herbarium of Nanjing Forestry University (NF).

### 4.2. DNA Extraction, PCR Amplification, and Sequencing

Total genomic DNA was extracted using the Plant Genomic DNA Kit #DP305 (Tiangen, Beijing, China). The quality of the extracted DNA was checked by 1% agarose gel electrophoresis, and the concentration and purity were evaluated using a NanoDrop^TM^ 2000 spectrophotometer (Thermo Scientific, Wilmington, DE, USA). Qualified DNA was stored at −80 °C. Two cpDNA fragments (*matK* and *trnS*-*trnG*) selected after screening were amplified with Tiangen 2 × Taq PCR Mix #KT201 (Tiangen, Beijing, China). PCR amplifications were conducted in a 25 μL reaction system containing 0.5 μL (10 μmol/L) of upstream and downstream primer, 12.5 μL of 2 × PCR Master Mix, 9.0 μL of ddH_2_O, and 2.5 μL of DNA template. The PCR procedure began with an initial denaturation at 94 °C for 5 min, followed by 30 cycles of 4 s denaturation at 94 °C, 30 s annealing at 55~65 °C, and 30 s extension at 72 °C, and a final extension at 72 °C for 5 min. After 1% agarose gels test, the qualified products were purified with a SanPrep Column PCR Product Purification Kit #B518141 (Sangon Biotech, Shanghai, China) and sequenced with Applied Biosystems^TM^ 3730XL Sequencer (Thermo Scientific, Santa Clara, CA, USA).

### 4.3. Genetic Diversity and Population Structure

The obtained sequencing data were processed using ContigExpress for peak map comparison, sequence validation, and assembly. PhyloSuite [49] was utilized to concatenate the two chloroplast fragments from each sample into a single cpDNA sequence, which was then aligned and analyzed in MEGA X [50]. DnaSP ver. 6 [51] was used to calculate the genetic diversity of each population, including haplotype number (*N*_h_), haplotype diversity (*H*_d_), nucleotide diversity (π), alongside estimating gene flow (*N*_m_) and genetic differentiation (*G_ST_*, *N_ST_*) between populations. Arlequin version ver. 3.5 [52] facilitated molecular variance analysis (AMOVA) with 1,000 non-parametric permutations to assess variances within and between populations and regions, and calculated the genetic differentiation among populations (*F_ST_*). A median-joining network for haplotype networks was constructed using PopART v. 1.7 [53] and haplotype geographical distribution maps were created using ArcGIS 10.2.

### 4.4. Phylogenetic Analysis and Divergence Time Estimation

To identify the phylogenetic relationship and divergence time of *O. settulatus*, four species from Oleaceae; *Chionanthus retusus* (HM751206.1, JX862836.1), *C. virginicus* (KP642959.1, JX862846.1), *Haenianthus salicifolius* (LN515430.1, JX862841.1), and *Comoranthus minor* (LN515430.1, JX862866.1) were chosen as outgroups. The phylogenetic relationships of haplotypes and outgroups were reconstructed using MEGA X based on the maximum parsimony (MP), maximum likelihood (ML), and Bayesian inference (BI) methods, respectively. The HKY + I + G model of substitution was selected by PartitionFinder of PhyloSuite [49]. Divergence time estimations of the cpDNA haplotypes lineages were performed using BEAST ver. 1.8.4 [54]. Lacking fossil records, three secondary calibration points for divergence time according to the diversification of Oleaceae were applied to calibrate node ages: Trib, Oleeae Crown, 46.66 Ma (node 1); Subtrib, Oleinae Crown, 33.78 Ma (node 2), and Genus *Chionanthus* and *Osmanthus*, 24.05 Ma (node 3) [55]. The data were analyzed using a relaxed log-normal clock model and the Yule process speciation model for the tree priors. A Markov chain Monte Carlo (MCMC) was run for 60 million generations with two parallel searches using four chains, each starting with a random tree. Trees were sampled every 1000 generations and the first 25% were discarded as burn-in. Tracer 1.7.2 [56] was used to inspect the convergence of the chains, ensure that effective sample size values for all parameters were greater than 200, and determine the substitution rates and the 95% highest posterior density (HPD). A maximum clade credibility tree was compiled with TreeAnnotator [57], with the posterior probability limit set to 0.5. FigTree v. 1.4.2 was used to check the result, and then the editing of the systematic tree was completed using tvBOT [58].

### 4.5. Demographic Analyses

Neutrality tests and mismatch distribution analysis (MDA) were carried out in Arlequin ver. 3.5 and DnaSP v. 6 to infer the historic demographic expansion events within the entire species. In the Neutrality tests, Tajima’s *D* [59] and Fu’s *Fs* [60] were estimated to detect population growth and expansion. MDA was performed to observe whether the expected value curve and observed value curve fit according to the distribution of base differences between different haplotypes. Moreover, the sudden expansion model was tested by the sum of squared deviation (*SSD*) between the expected and observed values and Harpending’s raggedness index (*H*_Rag_) [61].

## 5. Conclusions

Baoxing Osmanthus (*O. serrulatus*), a rare spring-flowering species of *Osmanthus*, is an important companion species in the evergreen broad-leaved forests of southwest China, which possess considerable research value. In this study, we conducted a phylogeographic investigation based on ten wild populations of *O. serrulatus*, aiming to provide evidence for the role of geographic and climatic factors in shaping its phylogeographic patterns. The results revealed only six haplotypes within the populations, indicating low genetic diversity, and significant genetic differentiation among populations, and low gene flow suggested that genetic variation primarily arises from inter-populations, showing a clear phylogeographic structure, while recent population expansions were absent. Human activities and habitat fragmentation, coupled with climate differences between regions and natural dispersal barriers such as mountains and valleys, have led to long-term isolation of *O. serrulatus* populations and intensified regional population differentiation. The species formation and phylogenetic diversity events of *O. serrulatus* reflect the significant influences of continuous climate changes and mountain uplift alternations during the late Pliocene and Pleistocene periods. The intra-specific haplotype diversity of *O. serrulatus* is likely shaped by Quaternary climate fluctuations. Our study clearly delineates the phylogeographic structure of *O. serrulatus*, infers its divergence time, analyses the impact of historical geological events on population dynamics, and reveals the influence of Quaternary climate oscillations on its distribution pattern. These findings provide important insights into the origin and evolution of southwestern evergreen broad-leaved forests, as well as significant implications for biodiversity conservation in the southwestern mountains.

## Figures and Tables

**Table 1:** Information on variation sites of chloroplast haplotypes of *O. serrulatus*.

Haplotype	Nucleotide Position				
	*matK*		*trnS-trnG*		
	396	699	1051	1098	1105
H1	G	C	A	A	T
H2	T	.	.	.	.
H3	T	.	.	C	.
H4	T	T	.	C	.
H5	.	.	.	C	.
H6	T	.	C	.	G

“.” represents the same base as the position of H1.

**Table 2:** Location and estimated diversity indexes of sampled *O. serrulatus* populations.

Populations	Locations	Sample Size	Longitude (E)	Latitude (N)	Elevation (m)	*H*_d_	π (×10^−3^)	Haplotypes (No. of Individuals)	*N*_h_
BCP	Jinkouhe, Leshan	12	103°09′	29°24′	1940	0.409	0.20	H1 (3)/H2 (9)	2
EMS	Emeishan, Leshan	18	103°20′	29°32′	2308	0.294	0.14	H3 (15)/H4 (3)	2
HZP	Hanyuan, Ya’an	26	102°33′	29°38′	2140	0.471	0.23	H3 (17)/H5 (9)	2
XYG	Hanyuan, Ya’an	18	102°42′	29°27′	2412	0.366	0.18	H3 (14)/H5 (4)	2
YCP	Hanyuan, Ya’an	18	102°34′	29°36′	2034	0.366	0.18	H3 (14)/H5 (4)	2
WZX	Lushan, Ya’an	7	103°02′	30°27′	2146	0.476	0.23	H3 (2)/H5 (5)	2
MPZ	Lushan, Ya’an	15	102°56′	30°22′	1318	0.343	0.17	H3 (12)/H5 (3)	2
DLS1	Baoxing, Ya’an	12	102°33′	30°25′	2080	0.712	0.43	H2 (4)/H3 (5)/H4 (3)	3
DLS2	Baoxing, Ya’an	11	102°32′	30°25′	2153	0.000	0.00	H3 (11)	1
XLXS	Dayi, Chengdu	11	103°10′	30°39′	2013	0.000	0.00	H6 (11)	1
mean						0.344	0.17		
All		148				0.590	0.47		

*H*_d_: haplotype diversity, π: nucleotide diversity, *N*_h_: numbers of haplotypes.

**Table 3:** Analyses of molecular variance (AMOVA) of *O. serrulatus* populations.

Source of Variation	d.f.	*SSD*	Variance Components	Percentage of Variation (%)	*F*_ST_	*G*_ST_ */* *N*_ST_	*N*_m_
Among groups	9	23.172	0.174 Va	81.23	0.812 (*p* < 0.05)	0.396/0.698 (*p* < 0.05)	0.11
Within populations	138	5.533	0.040 Vb	18.77			
Total	147	28.704	0.214				

d.f., degree of freedom; *SSD*, sum of squared differences.

**Table 4:** Neutrality test and mismatch distribution analysis of *O. serrulatus* populations.

Population	Tajima’s *D*	*p*	Fu’s *F_S_*	*p*	Demographic Expansion				Spatial Expansion			
					*SSD*	*p*	*H*_Rag_	*p*	*SSD*	*p*	*H*_Rag_	*p*
BCP	0.541	0.827	0.735	0.489	0.008	0.456	0.2	0.496	0.008	0.196	0.200	0.514
EMS	0.022	0.722	0.463	0.385	0.242	0.121	0.256	0.281	0.002	0.232	0.256	0.437
HZP	1.303	0.925	1.437	0.672	0.016	0.099	0.225	0.089	0.016	0.025	0.225	0.109
HYQ	0.488	0.804	0.796	0.515	0.005	0.426	0.206	0.417	0.005	0.206	0.206	0.447
HYX	0.488	0.782	0.796	0.501	0.005	0.408	0.206	0.427	0.005	0.199	0.206	0.455
WZX	0.559	0.85	0.589	0.459	0.017	0.270	0.229	0.615	0.017	0.208	0.229	0.622
LSD	0.235	0.767	0.597	0.443	0.003	0.486	0.216	0.382	0.003	0.25	0.216	0.416
DLS1	1.022	0.814	0.462	0.574	0.029	0.185	0.213	0.135	0.029	0.127	0.213	0.141
DLS2	0.000	1.000	0.000	N.A.	0.000	0.000	0.000	0.000	0.000	0.000	0.000	0.000
XLXS	0.000	1.000	0.000	N.A.	0.000	0.000	0.000	0.000	0.000	0.000	0.000	0.000
Mean	0.466	0.849	0.588	0.505	0.033	0.245	0.175	0.284	0.009	0.144	0.175	0.314

N.A.: The value is not available because there is only one haplotype in the population; *SSD*, sum of squared differences.

## Data Availability

All data generated or analysed during this study are included in this published article.
